# An Improved Weighted Partial Least Squares Method Coupled with Near Infrared Spectroscopy for Rapid Determination of Multiple Components and Anti-Oxidant Activity of Pu-Erh Tea

**DOI:** 10.3390/molecules23051058

**Published:** 2018-05-02

**Authors:** Ze Liu, Hua-lin Xie, Lin Chen, Jian-hua Huang

**Affiliations:** 1 Hunan Academy of Chinese Medicine, Changsha 410013, China; Ashelyze@163.com; 2 School of Pharmacy, Hunan University of Chinese Medicine, Changsha 410208, China; chenlin5202@126.com; 3 School of Chemical and Engineering, Yangtze Normal University, Chongqing 408100, China; hualinxie@163.com

**Keywords:** pu-erh tea, improved weight partial least squares, near infrared spectroscopy, quality assessment

## Abstract

*Background:* Pu-erh tea is a unique microbially fermented tea, which distinctive chemical constituents and activities are worthy of systematic study. Near infrared spectroscopy (NIR) coupled with suitable chemometrics approaches can rapidly and accurately quantitatively analyze multiple compounds in samples. *Methods:* In this study, an improved weighted partial least squares (PLS) algorithm combined with near infrared spectroscopy (NIR) was used to construct a fast calibration model for determining four main components, i.e., tea polyphenols, tea polysaccharide, total flavonoids, theanine content, and further determine the total antioxidant capacity of pu-erh tea. *Results:* The final correlation coefficients *R* square for tea polyphenols, tea polysaccharide, total flavonoids content, theanine content, and total antioxidant capacity were 0.8288, 0.8403, 0.8415, 0.8537 and 0.8682, respectively. *Conclusions*: The current study provided a comprehensive study of four main ingredients and activity of pu-erh tea, and demonstrated that NIR spectroscopy technology coupled with multivariate calibration analysis could be successfully applied to pu-erh tea quality assessment.

## 1. Introduction

China has a long history of using various kinds of tea, such as green, oolong, and black tea, which are produced with different processes [1]. Pu-erh tea is a unique microbially fermented tea, in which during the process of pile-fermentation, ripened pu-erh tea undergoes the enzymatic action of microorganisms in a humid environment [2]. Ripened pu-erh tea is mainly procuded from *Camellia sinensis var. assamica.* growing in Yunnan Province, located in the southwestern region of China [3]. As a health promoting beverage used for centuries, ripened pu-erh tea is of great value in the prevention of tumors, hyperlipidemia, hyperglycaemia, cardiovascular diseases, constipation, and other functions [4,5,6,7,8].

Bioactive compounds in ripened pu-erh tea, such as tea polyphenols, total flavonoids, tea polysaccharide, had been proven to have various pharmacological effects [8,9]. Some studies have demonstrated that polyphenols (TP) in ripened pu-erh tea had strong potential chemopreventive effects, especially in the prevention of cancer and cardiovascular diseases [10,11,12,13]. Total flavonoid (TF) in ripened pu-erh tea plays a significant role in lowering hyperlipidemia [14]. Tea polysaccharides showed pharmacological hypoglycemic activity [15,16], and could be used in the treatment of diabetes and other diseases [17,18].

Conventional approaches of determining teas’ properties are based on sensory evaluations [19]. Scientific studies on ripened pu-erh tea have been performed using chromatography methods, such as HPLC, and HPLC-DAD-MS [20,21,22]. However, previous analysis methods were complicated and time-consuming to some extent, in either analysis process or sample processing. Thus, it is essential to devise a rapid, nondestructive and cost-efficient quality evaluation methodology for ripened pu-erh tea.

Near-infrared spectroscopy (NIR) is a rapid, economic and nondestructive analytical technique. By combining it with suitable chemometrics methods, this technique has been widely applied in the quality control and determination of various chemical components in the agricultural, food and pharmaceutical industries [23,24]. One of the most widely used chemometrics algorithms for establishing calibration models is partial least squares (PLS), as this algorithm always provides good performance and flexible operation [25]. A classical PLS always operates by the non-linear iterative partial least squares (NIPALS) algorithm [26], which in a stepwise way extracts the PLS components by updating the weights, loadings, and scores. The weight describes the maximum covariance relationships between the variables and responses. When expected PLS components are extracted, the regression coefficients can be represented in terms of the weights and loadings. In recent studies, some improved PLS methods were proposed [27,28], such as interval PLS [29,30]. Li et al., proposed a sparse matrix weighted PLS method for human detection [31], where a lasso penalization was imposed on the weight vectors to produce a sparse solution for variable selection. Liu et al., proposed a regularized PLS method based on *l-2* norm [32]; *l-2* norm can keep the highly relevant variables down together by shrinking the regression coefficients toward zero. These modified methods showed some advantages over the raw PLS methods, and had better performances. Thus, much more attention has been focused on these improved multivariate calibration algorithms.

Therefore, in the current study, an improved weighted PLS method was developed based on the similarity principle; where the adjacent spectra were taken into account to adjust the weights of variables. then, this algorithm was further combined with a near infrared spectroscopy (NIR) method for simultaneously detecting the tea polyphenols, tea polysaccharide, total flavonoids, theanine content, and total antioxidant capacity of ripened pu-erh tea.

## 2. Results

### 2.1. Near Infrared Spectra

**Figure 1:**
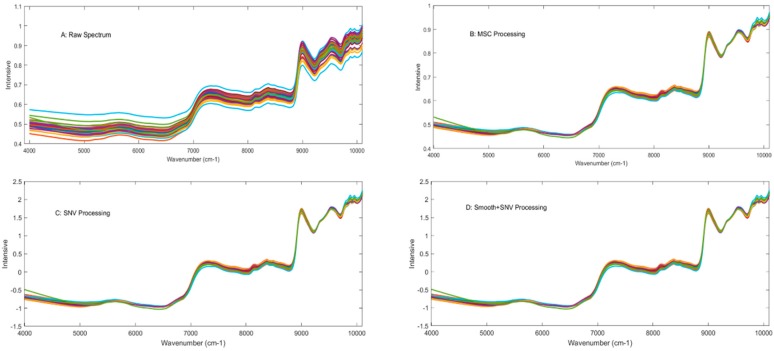
Raw NIR spectra of all samples (**A**) and processed with MSC, SNV, and “Smooth+SNV “methods, respectively (**B**, **C**, and **D**).

The NIR technique has been widely applied for simultaneously detecting various components of plants, foods, and other products, with many advantages, such as simple sample preparation, and rapid and nondestructive analysis. Original NIR spectra are similar and broad, consisting of many overlapping narrow bands corresponding to the different vibrational modes. Combination with some suitable spectral processing method is necessary for establishing the final calibration model. The raw NIR spectra of 40 pu-erh tea samples from wavenumbers 10,000 to 4000 cm^−1^, and spectra processed with different methods are exhibited in Figure 1A–D, respectively. Some strong signals are observed in the spectra, such as the signals from 5000 cm^−1^ to 5320 cm^−1^ caused by the second overtone vibration of the carbonyl group, and the combination of both C-O stretching and O-H deformation (~4750 cm^−1^), combination of both O-H stretching and first overtone of C-O deformation (~5200 cm^−1^).

### 2.2. NIR Calibration Model Establish

The tea polyphenols, tea polysaccharide, total flavonoids, theanine and total antioxidant capacity of ripened pu-erh tea were determined, and their spectral data were acquired by a FT-NIR spectrophotometer. Based on these experimental results, a quantitative model was constructed to rapidly and accurately determine these main components, and their antioxidant activities.

Forty Pu-erh tea samples were firstly classified into a calibration dataset (32 samples) and a prediction dataset (eight samples) by using the Kennard-Stone (K-S) algorithm with ratio 0.8. Detailed information about the samples in both groups is listed in Table 1.

In this section, we aimed to establish a reliable and accurate calibration model for these main components and the quantitative estimation of their anti-oxidant activities by using the PLS method and the proposed weighted PLS, respectively. During calibration model establishing, some spectra pre-processing methods were also optimized. All results were listed in Table 2 and Table 3, respectively.

As can be seen from these results, each component has its own suitable pre-processing method, with highest R^2^ and lowest RMSEP. In the raw PLS model, the suitable pre-processing methods for tea polyphenol, tea polysaccharide, and total flavonoid content were “smoothing combined with SNV”, SNV, and MCS method, respectively. The *R*^2^*_cal_* values for tea polyphenol, tea polysaccharide, and total flavonoid content were 0.9362, 0.9051 and 0.8955, respectively, while the RMSEC values of the calibration models for tea polyphenol, tea polysaccharide, and total flavonoid content were 0.2203, 0.0154, and 0.0987, respectively. The best pre-processing method for total antioxidant capacity is the SNV method, where the *R*^2^*_ca_*_l_ value and RMSEC value were 09179 and 0.0346, respectively (Table 2).

In Table 3, results were obtained based on the Weighted PLS model. The most suitable pre-processing methods for tea polyphenol and total flavonoid content were “smoothing combined with SNV” method. The suitable pre-processing methods for tea polysaccharide and total antioxidant capacity were “smoothing combined with SNV”, and MCS method, respectively. Furthermore, comparing with the raw PLS model, the weighted PLS could give a better prediction results for all the components (Table 3).

**Figure 2:**
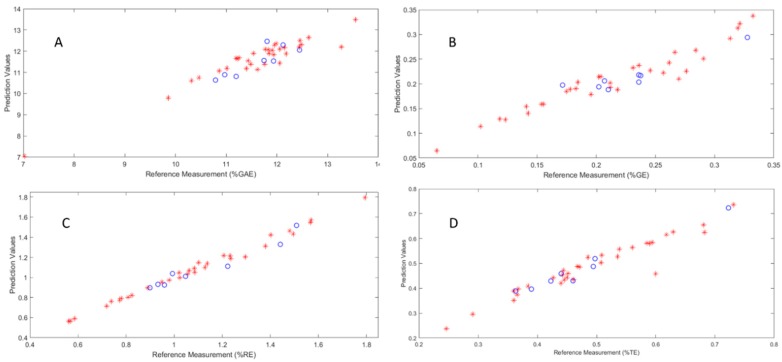
The calibration models for tea polyphenol (**A**), tea polysaccharide (**B**), total flavonoid (**C**) and antioxidant activities (**D**), respectively. Red circles and blue asterisks represent calibration and prediction test samples, respectively.

Based on these optimized experimental conditions, the calibration models were constructed, and then the prediction dataset was used to validate these models. These validation results are also listed in Table 2 and Table 3. The RMSEP values for tea polyphenols, tea polysaccharide, total flavonoids, and total antioxidant capacity calibration models were 0.3251, 0.0192, 0.1225 and 0.0587, respectively, while the R^2^ values for the tea polyphenols, tea polysaccharide, total flavonoids, and total antioxidant capacity calibration models were 0.8288, 0.8403, 0.8415 and 0.8682, respectively. The scatter plots of the calibration models for three main components and total antioxidant capacity, showing corrections between NIR prediction values and reference measurement, are plotted in Figure 2. These results suggest that the established models were accurate, and can schieve the aim of fast quantitative analysis of the main components and activities.

## 3. Discussion

In the current study, a weighted PLS method was proposed based on the similarities between variables and responses, and the distance method was adopted to estimate these similarities. The functional groups of substances always have relatively wide absorptions, thus adjacent wavelengths’ effects should be taken into accounts. In the proposed method, if a variable is similar to the response, it would be given a larger weight; this principle can be looked as like a variable selection process. That is why the proposed PLS has better performance than the raw PLS method. The authors think this idea can be further developed into a variable selection approach.

Furthermore, this weighted PLS method was used to determine tea polyphenols, tea polysaccharide, total flavonoids, theanine contents, and total antioxidant capacity of pu-erh tea samples by combining it with NIR spectrum data. The established calibration models showed good performances in rapidly and accurately predicting the main components and activity. The proposed method can be used to comprehensively evaluate the quality of pu-erh tea by combining the multiple active constituents and pharmacological activities simultaneously.

## 4. Materials and Methods

### 4.1. Sample Preparations and Reagents

Forty ripened pu-erh tea samples were collected from Pu’er city (Yunnan Province, China). These samples were powdered in a grinder, and passed through a 60-mesh sieve after drying. Powdered samples were labeled, and stored in plastic bags until chemical analysis, in a dry and shady place (25 °C).

Sodium carbonate (Na_2_CO_3_), sodium nitrite (NaNO_2_), aluminium chloride (AlCl_3_), sodium hydroxide (NaOH), phenol and concentrated sulfuric were purchased from Sinopharm (Guoyao Co. Ltd., Shanghai, China). 1,1-Diphenyl-2-picrylhydrazyl (DPPH), glucose, and Trolox were purchased from Sigma Company (St. Louis, MO, USA). Rutin and gallic acid used as standard materials were purchased from the China Food and Drug Inspection Institute (Beijing, China) and Hunan Institute for Food and Drug Control (Changsha, China), respectively. L-Theanine (99% purity) was purchased from Beijing Century Aroke Biotechnology Co. Ltd. (Beijing, China). HPLC-grade methanol (Hanbang Chemicals Co. Ltd., Suzhou, China), and phosphoric acid (Kermel, Tianjin, China) were also used. Deionized water was purified with a MillI-Q system (Millipore, Bedford, MA, USA).

### 4.2. Analysis Main Components and Their Anti-Oxidant Activity

The main compounds i.e., tea polyphenols, tea polysaccharide, total flavonoids, theanine content, are considered the most important anti-oxidants in tea. The DPPH assay is one of the most widely used methods in natural antioxidant studies. The absorbance of DPPH solutions was determined before and after reaction with plant antioxidants, and the absorbance decrease was used to estimate the reduction capability of DPPH radical [33]. Thus, the main active components in Pu-erh tea, and total antioxidant activity were determined (Supporting Information, Table S1).

#### 4.2.1. Extraction and Determination of Total Flavonoids

For the determination of total flavonoids, ultrasonic extraction was adopted using the method described in previous studies [34,35]. The solid-liquid ratio was controlled from 1 to 25 after adding 70% of ethanol, then sonication was implemented at 70 °C for 40 min. Consequently, the supernatant was diluted into a 50 mL volumetric flask after vacuum filtration. A 1 mg/mL standard solution of rutin was prepared as reference material. This rutin solution was then diluted to different concentrations (100, 200, 300, 400, 500 µg/mL) to calculate its calibration curve and verify its linearity. The content of total flavonoids was detected using the aluminum chloride method [36]. One mL pu-erh tea extract solution and 1 mL blank solution (70% ethanol in water) were added into two 10 mL test tubes, respectively. 5% sodium nitrite solution was added into the tubes equally, and after 6 min 0.5 mL of 1% aluminum chloride was added, and then the samples were shaken and stored for 6 min. The absorbency at a wavelength of 510 nm was measured after adding 4 mL of sodium hydroxide solution (1 mol/L). Finally, results were expressed as grams of rutin equivalent/100 g dry weight (g rutin/100 g tea extract). All samples were analyzed in triplicate.

#### 4.2.2. Extraction and Determination of Tea Polysaccharide

The determination of tea polysaccharide was performed by ultrasonic extraction referring to previous research [17]. After controlling the solid-liquid ratio at 1 to 15 by adding distilled water, sonication was implemented at 50 °C for 40 min. Next, the supernatant was diluted into a 25 mL volumetric flask after vacuum filtration. Ten mL of absolute ethanol were added to 2 mL of mixture, and then shaken. After leaving the solution to stand overnight, the solution was centrifuged (4000 rpm/min) for 30 min. The supernatant was discarded and the filtrate was diluted with water, and the dissolved filtrate was transferred into a 10 mL volumetric flask. A 1 mg/mL standard solution of glucose was prepared as reference solution. It was diluted to different concentrations within the range of 10, 20, 30, 40, 60, 80, 100 µm/mL to prepare a linear calibration curve. The content of tea polysaccharide was detected by the phenol-vitriol colorimetric method [37]. One mL of pu-erh tea extract solution and 1 mL of blank solution were added into two 10 mL test tubes, respectively. Then, 1.0 mL of 5% phenol solution was added into the tubes which then were shaken for 30 s. The mixture was shaken again for 30 s to ensure full mixing after adding 5.0 mL of concentrated sulfuric acid. the Absorbency was measured at the wavelength of 485 nm after successively heating the samples by putting them in a boiling bath for 20 min, and then cooling by putting them in a cold bath for 5 min. The content was expressed as grams of glucose equivalent/100g dry weight (g glucose/100g tea extract). All samples were run in triplicate.

#### 4.2.3. Extraction and Determination of Tea Polyphenols

According to national standard method [12], the optimal approach after optimizing the extraction conditions of tea polyphenols is as follows [38]. An accurately weighed, powdered sample (1.00 g) was transferred to a 100 mL conical flask; 70% ethanol (60 mL) was added; the sample was extracted at 70 °C for 35 min. Subsequently, the sample was cooled to room temperature and vacuum filtered. Filtrates were diluted to volume in a 100 mL volumetric flask using ethanol-water solution; then stored in a refrigerator at 4 °C for tea polyphenols determination. For determining the tea polyphenols, the Folin-Ciocalteu reagent was applied, which has been widely acknowledged as a national standard method for the determination of polyphenol substances. Gallic acid was applied in this determination and the detailed procedure is as follows. The diluted (10 times) Folin-Ciocalteu reagent (5 mL) was added and then samples were shaken. The tubes were allowed to equilibrate at ambient temperature (25 ± 0.2 °C) for 4 min, and then 4 mL of 7.5% (*w*/*v*) sodium carbonate was added with a 20 s vortexing step. Finally we measured the absorbency under the maximum absorption wavelength of 747 nm after storing in a cool and dark place for 60 min. The TPC was expressed as grams of gallic acid equivalent (GAE)/100 g material using a gallic acid calibration plot, where the standard curve of gallic acid ranged from 10–80 μg/mL (R^2^ = 0.9998). All samples were tested in triplicate.

#### 4.2.4. Preparation and Determination of Theanine Content

Powdered sample (1.0 g) was extracted with water (50 mL) in a 100 mL conical flask. The mixture was sonicated for 30 min at the extraction temperature of 60 °C, and the supernatant was filtered under vacuum after cooling to room temperature. Subsequently, the solution was filtered through a 0.45 μm membrane before HPLC analysis. In order to obtain the linear range of quantification, a stock standard solution of L-theanine (6 mg/mL) was prepared with water and stored at 4 °C, and finally the standard curve of L-theanine in the range from 0.05–2 mg/mL was determined. 

To determine the content of L-theanine, a RP-HPLC method was performed by using an Agilent 1260 series instrument (Palo Alto, CA, USA). A reverse-phase Agilent TP-C18 column (4.6 mm × 250 mm, 5 μm) was used. The column temperature was maintained at 30 °C. The injected volume was 10 μL and the flow rate was 1.0 mL/min. The mobile phases which were used for gradient HPLC were (A) 0.1% phosphoric aqueous acid and (B) methanol. The detailed gradient was given as follows: 2% B at 0–8 min, 2–95% B at 8–10 min, 95% B at 10–20 min, and 95–2% at 20–25 min. The DAD detection was performed at 210.4 nm. The determination of L-theanine in tea samples is calculated according to the chromatographic peak area of the L-theanine standard substance.

#### 4.2.5. TAA-DPPH Assay

Total antioxidant activity in pu-erh tea was assessed using a 1,1-diphenyl-2-picrylhydrazyl (DPPH) radical scavenging assay [39]. A 2.5 mmol/mL standard solution of Trolox was prepared as a reference. The standard solution was diluted to different concentrations, which showed linearity within the range of 58.5938, 49.0196, 39.3701, 29.6443, 19.8413, 9.9602 µmol/mL. A five µL extracted sample solution and blank (pure methanol) solution were added into a 96-well plate, respectively. Every sample was analyzed in duplicate adding equal amounts of DPPH solution with methanol. the Absorbency was determined at an absorption wavelength of 515 nm after storing in a cool and dark place at room temperature for 30 min [40].

The inhibition ratio was calculated according to the following formula:

S (%) = (A − B)/A × 100%

where S: clearance rate; A: absorbance value of blank solution; B: absorbance value of sample solution.

### 4.3. NIR Spectroscopy Measurements

The NIR spectra were obtained in diffuse reflectance mode using an Antaris II FT-NIR spectrophotometer (Thermo EIectron Co., Waltham, MA, USA) equipped with an integrating sphere. The spectra (32 scans, 8 cm^−1^ resolution, 4000 to 10,000 cm^−1^, and 1557 points/spectrum) were collected in the log (1/R) mode (R = the reflectance value). Dry Pu-erh sample (0.5 g) was added into the sample cup, each sample was measured three times, and the mean of the three spectra was used for further statistical analysis. The temperature was kept at 25 °C.

### 4.4. Weighted Partial Least Squares Model

The partial least squares regression (PLSR) method is widely used to establish calibration models. PLS models can decompose both independent variable (*X*) and dependent variable (*Y*), simultaneously, and aim to find the latent variables in *X* to predict the latent variables in *Y*.

Let X be the *m* × *p* data matrix, whose rows and columns correspond to samples and variables (spectral intensity in correspond wavelength), respectively, and *Y* matrix is a *m* × *k* response vector. PLS aims to find a weight vector $w\in R^{p}$, which can maximize the covariance between the independent variables *X*, and the corresponding dependent variable *Y*. *X* and *Y* can be further decomposed by the PLS method as follows:

(1) $$X=TP^{\prime }+E\;Y=UQ^{\prime }+F$$

where the latent variables *T*, *U* are extracted from *X*, *Y*, and, *P*, *Q* are the score matrices. *E* and *F* are the residual matrices. The desired weight vector can be represented as:

(2) $$\hat{w}=\frac{X^{T}y}{∥X^{T}y∥}$$

From Equation (2), we can see that the weight vector $\hat{w}$ reflects the relationships between the response *y* and each *X* variable *X*_1_, …, *X_p_*. Therefore, the computation of weight directly affects the final PLS model. Furthermore, if a variable *X_i_* is very similar to the response *Y*, it should be assigned a large weight value *W_i_*. There are many approaches to estimate the similarities between variables and response; among them, distance is a simple and effective mode.

Based on this idea, we proposed a new weight partial least squares to establish the quantitative model, in which the weights of variables were adjusted according to the distance between the variable and response. Instead of using the single variable to calculate the distance, the adjacent variables’ effects were also taken into account (as the functional groups always have absorptions within relatively short wavelength bands). By using the adjusted distance, the adjacent wavelengths’ effects can be balanced, these can be defined as:

(3) $$\begin{array}{l} D1=dist(y,X_{i})={\left(∥y-x_{i}∥\right)}^{T}\\ D2=dist(y,X_{\left(i-l,i+l\right)})={\left(∥y-x_{1}∥,\ldots ,∥y-x_{p}∥\right)}^{T}\\ D=D1+D2 \end{array}$$

Using the following regularization framework:

(4) $$\hat{w}=\arg\underset{w}{\min}\left\{∥y-Xw{∥}^{2}+\delta ∥Dw{∥}^{2}\right\}$$

where δ is the regularization parameter, and *D* is a diagonal regularization matrix. The first term in Equation (4) ensures that the solved weight vector keeps the relations between *Y* and *X,* while the second term penalizes the variables with a large distance to the response; moreover, if these adjacent wavelength brands are also far from the response, this will aggravate the penalties i.e., lower weights will be given to these variables. In the new algorithm, the regularization parameter δ was pre-set to 1, the width of wavelength *l* for each side was also pre-set to 5 empirically. For the first and last *l* variables, *i* + 10 and *i* − 10 wavelengths were used to calculate the distances, respectively.

## Figures and Tables

**Table 1:** Samples in calibration dataset and prediction dataset.

Indexes	Calibration Dataset			Prediction Dataset		
	Concentration Range	Mean Value	Standard Deviation	Concentration Range	Mean Value	Standard Deviation
Tea polyphenol	7.02–13.55	11.59	1.13	10.79–13.56	11.62	0.58
Tea polysaccharide	0.065–0.33	0.21	0.069	0.17–0.32	0.22	0.045
Total flavonoid	0.568–1.798	1.072	0.31	0.93–1.44	1.12	0.23
Theanine content	5.32–19.41	8.809	2.74	6.03–13.84	10.24	2.79
Antioxidant activities	0.25–0.73	0.49	0.15	0.38–0.72	0.47	0.10

**Table 2:** Results for calibration model with different spectrum pre-processing based on Raw PLS algorithm.

Indexes	Evaluations	Raw	MSC	SNV	Smooth	Smooth + SNV	Smooth + MSC
Tea polyphenol	RMSEC	0.3948	0.2515	0.2324	0.3557	0.2203	0.2441
	*R* ^2^ *_cal_*	0.8771	0.9062	0.9313	0.8965	0.9362	0.9113
	RMSEP	0.4306	0.4026	0.3635	0.4185	0.3406	0.3985
	*R* ^2^ *_pre_*	0.7484	0.7800	0.8207	0.7691	0.8239	0.7845
Tea polysaccharide	RMSEC	0.0252	0.0179	0.0154	0.0198	0.0155	0.0179
	*R* ^2^ *_cal_*	0.8259	0.8997	0.9051	0.8763	0.9025	0.8913
	RMSEP	0.0428	0.0315	0.0216	0.0376	0.0265	0.0353
	*R* ^2^ *_pre_*	0.7281	0.8094	0.8217	0.7855	0.8203	0.8052
Total Flavonoid	RMSEC	0.1205	0.0987	0.1028	0.1126	0.1005	0.1023
	*R* ^2^ *_cal_*	0.8363	0.8955	0.8869	0.8579	0.8829	0.8932
	RMSEP	0.1521	0.1495	0.1551	0.1598	0.1518	0.1505
	*R* ^2^ *_pre_*	0.7561	0.8283	0.8174	0.8051	0.8123	0.8215
Theanine content	RMSEC	1.093	1.106	0.9705	1.183	1.135	0.9909
	*R* ^2^ *_cal_*	0.8756	0.8614	0.8686	0.8705	0.8579	0.8630
	RMSEP	1.167	1.118	1.093	1.128	1.125	1.130
	*R* ^2^ *_pre_*	0.8101	0.8254	0.8638	0.8046	0.8200	0.8574
Antioxidant Activities	RMSEC	0.0541	0.0425	0.0346	0.0458	0.0326	0.0456
	*R* ^2^ *_cal_*	0.8259	0.8875	0.9179	0.8442	0.9126	0.8824
	RMSEP	0.0862	0.812	0.0634	0.0816	0.0687	0.0693
	*R* ^2^ *_pre_*	0.7864	0.8196	0.8582	0.7954	0.8556	0.8423

**Table 3:** Results for calibration model with different spectrum pre-processing based on Weighted PLS algorithm.

Indexes	Evaluations	Raw	MSC	SNV	Smooth	Smooth + SNV	Smooth + MSC
Tea polyphenol	RMSEC	0.4087	0.2970	0.2685	0.3715	0.2241	0.2436
	*R* ^2^ *_cal_*	0.8647	0.9279	0.9410	0.88775	0.9589	0.9514
	RMSEP	0.4053	0.3264	0.4068	0.4249	0.4532	0.3251
	*R* ^2^ *_pre_*	0.7669	0.8190	0.7523	0.7625	0.7726	0.8288
Tea polysaccharide	RMSEC	0.0214	0.0188	0.0184	0.0223	0.0163	0.0192
	*R* ^2^ *_cal_*	0.8428	0.8782	0.8832	0.8300	0.9084	0.8736
	RMSEP	0.03281	0.0234	0.0216	0.02761	0.0192	0.0216
	*R* ^2^ *_pre_*	0.7681	0.8021	0.8217	0.7855	0.8403	0.8152
Total Flavonoid	RMSEC	0.1152	0.1001	0.0991	0.1195	0.0956	0.0849
	*R* ^2^ *_cal_*	0.8488	0.8839	0.8879	0.8374	0.8960	0.9177
	RMSEP	0.1896	0.1333	0.1479	0.1592	0.1528	0.1225
	*R* ^2^ *_pre_*	0.7861	0.8283	0.8174	0.7967	0.8023	0.8415
Theanine content	RMSEC	1.093	0.8049	0.7453	0.8850	0.8421	0.7745
	*R* ^2^ *_cal_*	0.8756	0.9096	0.9225	0.8908	0.9011	0.9163
	RMSEP	1.167	1.082	1.095	1.331	1.317	1.309
	*R* ^2^ *_pre_*	0.8109	0.8283	0.8537	0.7821	0.7853	0.7881
Antioxidant Activities	RMSEC	0.0378	0.0319	0.0300	0.0420	0.0355	0.03702
	*R* ^2^ *_cal_*	0.8936	0.9245	0.9333	0.8690	0.9066	0.8985
	RMSEP	0.0782	0.0678	0.0587	0.0862	0.0652	0.0693
	*R* ^2^ *_pre_*	0.8211	0.8556	0.8682	0.8054	0.8596	0.8329

## Supplementary Materials

The following are available online. Figure S1: Chromatography of Theanine standard and one tea sample, Table S1: The main components of 40 pu-erh tea samples, and their anti-oxidant activity.
