# Supplementary material for: An Improved Weighted Partial Least Squares Method Coupled with Near Infrared Spectroscopy for Rapid Determination of Multiple Components and Anti-Oxidant Activity of Pu-Erh Tea
Source: Molecules. 2018 May 2;23(5):1058. doi: 10.3390/molecules23051058 (PMC6102534; doi:10.3390/molecules23051058)
Supplement: Supplementary file 1 [file molecules-23-01058-s001.zip › molecules-285028-SI.pdf]

Supporting Information

An Improved Weighted Partial Least Squares Method Coupled with Near Infrared Spectroscopy for Rapid Determination of Multiple Components and Anti-Oxidant Activity of Pu-Erh Tea

Ze Liu [1,2,†], Hua-lin Xie [3,†], Lin Chen [2] and Jian-hua Huang [1,2,*]

[1] Hunan Academy of Chinese Medicine, Changsha 410013 , China; Ashelyze@163.com
[2] School of Pharmacy, Hunan University of Chinese Medicine, Changsha 410208, China; chenlin5202@126.com
[3] School of Chemical and Engineering, Yangtze Normal University, Chongqing 408100, China; hualinxie@163.com
* Correspondence: huangjianhua1985@163.com; Tel.: +86-731-8880-7174
† The first two authors have contributed equally to this article.

Analysis of theanine contents by an HPLC method

A robust HPLC reference method was prior established to quantitatively analyze theanine contents in these samples. Figure S1 shows the HPLC profiles of a theanine standard and one tea sample.

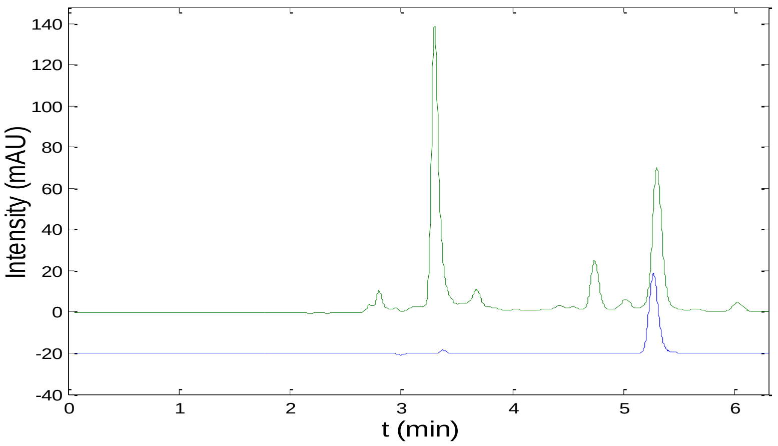

Fig s1: Chromatography of Theanine standard and one tea sample.

The methodology parameters and the calibration curves of the HPLC method were investigated and optimized and listed as follows:

Method validation

Linearity, limits of quantification and detection

The linearity of the theanine analysis was assessed using five different concentrations (0.05 – 2.0 mg/mL), and verified using the optimal chromatographic conditions. Linear regression was performed to determine the equation of the line, giving the linear equation y=2167.6x+32.2, correlation coefficient r = 0.9995. The limit of detection (LOD), and limit of

quantification (LOQ) values were defined as the signal-to-noise ratio (S/N) of 3 and 10, LOD and LOQ were 9.34375 μg/mL and 31.14580 μg/mL, respectively.

Precision, repeatability and stability

Intra-day variations were utilized to assess the precision of the developed method. Repeatability was evaluated by analyzing one sample using above described procedure within a single day. For evaluation of sample stability, the sample was tested at 0, 2, 4, 6, 8, 12 and 24 h. The relative standard deviation (RSD) values of precision, repeatability, stability, and recovery 1.16%, 1.71%, 0.40% and 2.31%, respectively.

Table S1: The main components of 40 pu-erh tea samples, and their anti-oxidant activity.

Sample No.	Tea polyphenols	Tea Polysaccharide	Total Flavonoids	Theanine content	Antioxidant Activities
1	24.89	0.12	1.57	8.818	0.60
2	24.91	0.18	1.30	6.594	0.59
3	23.83	0.07	1.02	8.835	0.44
4	23.61	0.21	0.93	7.904	0.36
5	22.80	0.12	1.02	9.649	0.46
6	22.98	0.27	0.82	7.587	0.37
7	23.98	0.17	1.13	8.770	0.47
8	22.87	0.16	0.77	8.948	0.37
9	23.90	0.20	1.06	6.722	0.45
10	23.07	0.10	0.74	5.915	0.45
11	22.39	0.23	1.24	10.74	0.36
12	23.24	0.27	1.08	7.095	0.45
13	23.68	0.22	0.56	7.062	0.25
14	23.70	0.14	0.98	9.390	0.47
15	22.03	0.24	1.23	8.589	0.56
16	24.37	0.17	0.78	10.78	0.36
17	24.89	0.20	1.05	7.779	0.46
18	23.87	0.26	1.51	7.242	0.44
19	23.49	0.33	0.96	5.325	0.39
20	22.51	0.18	1.14	8.068	0.49
21	24.11	0.25	0.57	7.376	0.29
22	21.72	0.26	0.81	10.86	0.43
23	22.44	0.15	1.10	5.935	0.59
24	25.26	0.29	1.21	7.531	0.62
25	23.52	0.18	1.24	8.948	0.44
26	24.24	0.24	1.22	11.53	0.49
27	21.94	0.21	0.90	13.83	0.42
28	20.92	0.20	0.89	7.594	0.38
29	24.30	0.21	1.40	14.23	0.58
30	22.39	0.33	1.51	12.85	0.50

Table S1. *Cont.*

31	27.10	0.24	1.44	10.95	0.72
32	24.96	0.21	1.38	11.55	0.68
33	20.64	0.24	1.50	11.96	0.63
34	19.72	0.20	0.59	19.41	0.73
35	21.58	0.32	0.99	11.05	0.44
36	23.54	0.28	1.48	10.19	0.51
37	24.11	0.31	1.57	7.350	0.54
38	26.54	0.14	0.81	8.483	0.68
39	23.88	0.28	0.95	6.030	0.54
40	14.05	0.32	1.09	6.812	0.51
